# Association of Sedentary Behavior With Anxiety, Depression, and Suicide Ideation in College Students

**DOI:** 10.3389/fpsyt.2020.566098

**Published:** 2020-12-11

**Authors:** Liyuan Jiang, Yong Cao, Shuangfei Ni, Xiang Chen, Minxue Shen, Hongbin Lv, Jianzhong Hu

**Affiliations:** ^1^Department of Spine Surgery, Xiangya Hospital, Central South University, Changsha, China; ^2^Key Laboratory of Organ Injury, Aging and Regenerative Medicine of Hunan Province, Changsha, China; ^3^Department of Dermatology, Xiangya Hospital, Central South University, Changsha, China; ^4^Department of Social Medicine and Health Management, Xiangya School of Public Health, Central South University, Changsha, China; ^5^Department of Sports Medicine, Xiangya Hospital, Central South University, Changsha, China

**Keywords:** sedentary behavior, emotional distress, college student, sleeping quality, mediation analysis

## Abstract

**Objectives:** To investigate the association of sedentary behavior with anxiety, depression, and suicide ideation in multi-centered college students in China.

**Methods:** This was a cross-sectional study of the first-year college student population. The students underwent a questionnaire survey inquiring about sedentary behavior (hours per day) and physical activity (minutes per week) during the past year. Anxiety, depression, and sleep quality were measured by the Generalized Anxiety Disorder Scale (GAD-2), the Patient Health Questionnaire (PHQ-2), and the Pittsburgh Sleep Quality Index (PSQI), respectively. Mixed models were used to estimate the associations, and adjusted odds ratios (AORs) were presented as the effect size. Mediation effect analysis was conducted to test the mediation effect of PSQI.

**Results:** A total of 28,298 participants (response rate: 82%) completed the survey and were included in the final analyses. Crude and adjusted estimates consistently showed that both sedentary behavior and physical activity were significantly associated with mental illnesses. Sedentary behavior was positively associated with anxiety, depression, and suicidal behavior in a dose-response manner (AOR: 0.54–0.24; ≥7 h/day as reference), independent from the effect of physical activity (AOR: 0.78–0.41; no physical activity as reference). The association of sedentary behavior with mental health was partly mediated by sleep quality (25–71%).

**Conclusions:** There is an independent dose-response association of sedentary behavior with mental well-being among college students in China, and this association may be partially attributable to impaired sleep quality. Attention should be drawn and actions should be taken by college educators and mental health providers.

## Introduction

The concerns regarding mental health increased in the college population (1). College students with mental illness usually experience impaired self-esteem, stigmatization, bullying, and family and social relationship failures (2). Various behavioral-level risk factors have been shown to contribute to emotional distress, and the increasingly less physical activity among adolescents over decades is one of them.

Physical inactivity has been one of the significant public health concerns of the twenty-first century due to its increased trends and its multiple adverse effects on human health (3). It is reported that 31.1% of adults (15 years or older) are physically inactive worldwide, which is more common in high-income countries and the elderly population (4). The reverse association of physical activity with emotional distress has been well established in the past decades (5–7). An increasing body of literature indicates that physical activity can be beneficial on mental health among adolescents and young adults (8–10).

Sedentary behavior (11), defined as a status that a person is in a sitting, reclining, or lying posture, has been gradually common along with less physical activity among adolescents (12). Although frequently mutually associated, sedentary behavior is not physical inactivity by a different name (13). As a fact, accumulating epidemiological evidence suggests physical activity by itself is insufficient to eliminate the risks of sedentary behavior. However, compared to physical activity and emotional distress, the majority of the previous studies detected the association of sedentary behavior and emotional distress together with physical activity (14). Most of the researchers viewed that sedentary behavior and physical activity are interactively but not independently influencing the illness of the mental health of adolescents (15).

In our study, we aim to detect the association of sedentary behavior with emotional distress through a multi-centered college student population in China and to explore their possible mediators.

## Methods

### Study Design

This was a cross-sectional study. Data collected from 2017 to 2018 were used for analysis. In 2017, the pilot study was conducted in a university in Changsha, China (16, 17). In 2018, the main study was conducted in five universities in different regions of China (18). All first-year college students that consented to participate received an online questionnaire survey after their enrollment to the universities. The questionnaire survey was organized by the departments of student affairs of the universities. The medical ethics committee approved this study of Xiangya Hospital, Central South University (approval number: 201709993). All participants were aged above 16, and written informed consent was obtained from all the students before the investigation.

### Exposure Variables

Sedentary behavior (hours per day) and physical activity (minutes per week) during the past year were measured by a self-reported questionnaire. Sedentary behavior was defined as sitting behaviors beyond class time, including using a smartphone or computer and watching TV. Sitting time included four categories: <1, 1–2.9, 3–6.9, and ≥7 h/day. Physical activity was measured as the product of frequency per week and duration per time and was grouped into five categories: no physical activity, 1–59, 60–179, 180–419, and ≥420 min/week.

### Covariates

Potential confounders included age, gender, annual family income (socioeconomic status indicator), parental education level, ethnicity (Han vs. other), the region of hometown, body mass index (BMI), smoking, and alcohol drinking. BMI was calculated as weight (kg)/height^2^ (m^2^); height and weight were measured by nurses using standardized methods during the health examination. Education level and marital status were not included. Sleep quality, measured by the Pittsburgh Sleep Quality Index (PSQI) (19), was considered as a mediator in the association of sitting with emotional well-being.

### Outcome Assessment

Anxiety and depression were measured by the 2-item Generalized Anxiety Disorder (GAD-2) Scale and two-item Patient Health Questionnaire (PHQ-2), respectively, inquiring about the symptoms of anxiety and depression in the past month. GAD-2 and PHQ-2 were then dichotomized by cutoff ≥3 according to previous studies showing good sensitivity and specificity (20, 21).

Suicide ideation was determined by a single question “Did you have ideation of killing yourself in the past month.” The response included “Never,” “Couple of days,” “Half of the time,” and “Nearly every day.”

### Statistical Analyses

Analyses were performed using SAS software version 9.2 (SAS Institute, Inc., Cary, North Carolina, United States). Continuous data were presented as the mean ± standard deviation, and between-group differences were tested using analysis of variance (ANOVA). Categorical data were presented as number (%), and between-group differences were tested using the chi-square test.

To consider the potential center effect, mixed models (student as a level-1 unit and university as a level-2 unit) with proper link function (identity for the continuous outcome and logit for binary outcome) were used to estimate the effects of sedentary behavior and physical activity on anxiety, depression, and suicide ideation, adjusting for level-1 and level-2 confounders. The null model (with no independent variable) was used to detect the center effect at the university level, and intra-cluster correlation coefficients (ICCs) were reported to describe the center effect. Adjusted odds ratios (AORs) with 95% confidence intervals (CIs) were used to demonstrate effect size. We examined the joint association between physical activity and sedentary behavior by deriving a combined variable with 20 groups, where the combined highest sitting (<1 h/day) and no physical activity. We also examined the modification effect between physical activity and sitting by introducing the interaction term in the model. Mediation effect analysis was conducted to explore the potential effect of sleep quality as a mediator using the R package “mediation.” Sensitivity analysis was performed by excluding the data collected in the pilot study in 2017.

## Results

### Characteristics of Participants

A total of 34,481 students from five universities underwent the health examination, and 28,298 (82%) completed the questionnaire and included in the final analysis. The characteristics (age and gender) of the subjects were not statistically different from the rest of the students. Characteristics of the subjects are shown in Table 1, stratified by the time of daily sedentary behavior. The mean age was 18.3 ± 0.8 years, and 53.5% of the participants were male. Sedentary behavior showed a strong inverse association with physical activity. Descriptive analysis showed that sedentary behavior was significantly associated with higher prevalence rates of anxiety, depression, and suicide ideation. In contrast, physical activity showed an inverse association (Table 2).

**Table 1:** Characteristics of participants by sedentary behavior.

**Variable**	**Category**	**Total**	**Sedentary behavior (h/day)**			
			** <1**	**1–2.9**	**3–6.9**	**≥7**
**Cluster-level variables**						
Year of enrolment	2017	8,226 (29.1)	680 (30.0)	4,044 (30.8)	3,295 (27.9)	207 (19.4)
	2018	20,072 (79.9)	1,588 (70.0)	9,088 (69.2)	8,536 (72.1)	860 (80.6)
Location of university	Changsha	13,224 (46.7)	1,076 (47.4)	6,345 (48.3)	5,417 (45.8)	386 (36.2)
	Wuhan	5,588 (19.8)	426 (18.8)	2,696 (20.5)	2,249 (19.0)	217 (20.3)
	Xiamen	4,196 (14.8)	272 (12.0)	1,703 (13.0)	2,007 (17.0)	214 (20.1)
	Urumchi	2,912 (10.3)	320 (14.1)	1,374 (10.5)	1,111 (9.4)	107 (10.0)
	Hohhot	2,378(8.4)	174 (7.7)	1,014 (7.7)	1,047 (8.8)	143 (13.4)
**Individual-level variables**						
Region of hometown	North	4,583 (16.2)	362 (16.0)	2,084 (15.9)	1,930 (16.3)	207 (19.4)
	Northeast	1,052 (3.7)	109 (4.8)	503 (3.8)	393 (3.3)	47 (4.4)
	East	6,197 (21.9)	498 (22.0)	2,824 (21.5)	2,647 (22.4)	228 (21.4)
	Central	6,420 (22.7)	445 (19.6)	2,955 (22.5)	2,790 (23.6)	230 (21.6)
	South	2,202 (7.8)	134 (5.9)	993 (7.6)	995 (8.4)	80 (7.5)
	Southwest	2,868 (10.1)	198 (8.7)	1,325 (10.1)	1,236 (10.5)	109 (10.2)
	West	4,976 (17.6)	522 (23.0)	2,448 (18.6)	1,840 (15.5)	166 (15.5)
Age (year)		18.3 ± 0.8	18.4 ± 0.9	18.3 ± 0.8	18.3 ± 0.8	18.3 ± 0.8
Body mass index (kg/m^2^)		21.2 ± 3.5	21.3 ± 3.5	21.3 ± 3.6	21.2 ± 3.5	21.3 ± 3.6
Gender	Male	15,141 (53.5)	1,285 (56.7)	6,982 (46.1)	6,341 (53.6)	533 (49.9)
	Female	13,157 (46.5)	983 (43.3)	6,150 (46.8)	5,490 (46.4)	534 (50.1)
Ethnicity	Han	23,398 (82.7)	1,765 (77.8)	10,834 (82.5)	9,926 (83.9)	873 (81.8)
	Other	4,900 (17.3)	503 (22.2)	2,298 (17.5)	1,905 (16.1)	194 (18.2)
Annual family income (CNY)	<10,000	2,929 (10.3)	331 (14.6)	1,326 (10.1)	1,146 (9.7)	126 (11.8)
	10,001–30,000	6,232 (22.0)	527 (23.2)	2,860 (21.8)	2,598 (22.0)	247 (23.1)
	30,001–50,000	4,859 (17.2)	342 (15.1)	2,232 (17.0)	2,095 (17.7)	190 (17.8)
	50,001–99,999	6,331 (22.4)	457 (20.1)	2,933 (22.3)	2,712 (22.9)	229 (21.5)
	100,000–199,999	5,736 (20.3)	432 (19.1)	2,737 (20.8)	2,367 (20.0)	200 (18.7)
	≥200,000	2,211 (7.8)	179 (7.9)	1,044 (8.0)	913 (7.7)	75 (7.0)
Smoking	No	27,906 (98.6)	2,235 (98.5)	12,966 (98.7)	11,665 (98.6)	1,040 (97.5)
	Yes	392 (1.4)	33 (1.5)	166 (1.3)	166 (1.4)	27 (2.5)
Alcohol drinking	No	26,990 (92.4)	2,182 (96.2)	12,615 (96.1)	11,207 (94.7)	986 (92.4)
	Yes	1,308 (6.2)	86 (3.8)	517 (3.9)	624 (5.3)	81 (7.6)
Physical activity (min/week)	No	8,436 (29.8)	571 (25.2)	3,286 (25.0)	4,080 (34.5)	499 (46.8)
	1–59	3,367 (11.9)	329 (14.5)	1,700 (12.9)	1,256 (10.6)	82 (7.7)
	60–179	6,584 (23.3)	572 (25.2)	3,327 (25.3)	2,525 (21.3)	160 (15.0)
	180–419	7,541 (26.6)	546 (24.1)	3,706 (28.2)	3,087 (26.1)	202 (18.9)
	≥ 420	2,370 (8.4)	250 (11.0)	1,113 (8.5)	883 (7.5)	124 (11.6)

**Table 2:** Distribution of anxiety, depression, and suicide ideation in sedentary behavior and physical activity categories.

**Outcome**	**Sedentary behavior (h/day)**					**Physical activity (min/week)**					
	** <1**	**1–2.9**	**3–6.9**	**≥7**	***P***	**No**	**1–59**	**60–179**	**180–419**	**≥420**	***P***
Anxiety^a^	116 (5.1)	621 (4.7)	960 (8.1)	188 (17.6)	<0.001	880 (10.4)	180 (5.4)	360 (5.5)	356 (4.7)	109 (4.6)	<0.001
Depression^b^	116 (5.1)	614 (4.7)	863 (7.3)	155 (14.5)	<0.001	762 (9.0)	183 (5.4)	324 (4.9)	367 (4.9)	112 (4.7)	<0.001
Suicide ideation	64 (2.8)	300 (2.3)	430 (3.6)	80 (7.5)	<0.001	397 (4.7)	80 (2.4)	185 (2.8)	157 (2.1)	55 (2.3)	<0.001

*GAD-2, Two-Item Generalized Anxiety Disorder Scale. PHQ-2, Two-Item Patient Health Questionnaire*.

a *Defined as GAD-2 score ≥3*.

b *Defined as PHQ-2 score ≥3*.

### Center Effect

Null models showed that the ICCs (proportion of level-2 covariance) varied from 3 to 4% for continuous variables, and 12 to 18% for categorical variables (Supplementary Table 1), indicating the center effect of the university and the necessity of using mixed models.

### Independent Effect

Crude and adjusted estimates consistently showed that less sedentary behavior and more physical activity were associated with lower prevalence rates of anxiety, depression, and suicide ideation (Supplementary Table 2). Compared with sedentary behavior ≥7 h/day, sitting time between 1 and 2.9 h/day had the largest effect size for anxiety (AOR = 0.28, 95% CI: 0.23–0.34, *P* < 0.001), depression (AOR = 0.34, 95% CI: 0.28–0.42, *P* < 0.001) and suicide ideation (AOR = 0.36, 95% CI: 0.28–0.47, *P* < 0.001). Physical activity was also independently associated with anxiety, depression, and suicide ideation with effect size ranging from 0.47 to 0.78, after adjusting for sedentary behavior and potential confounders.

### Joint and Modification Effect

**Figure 1:**
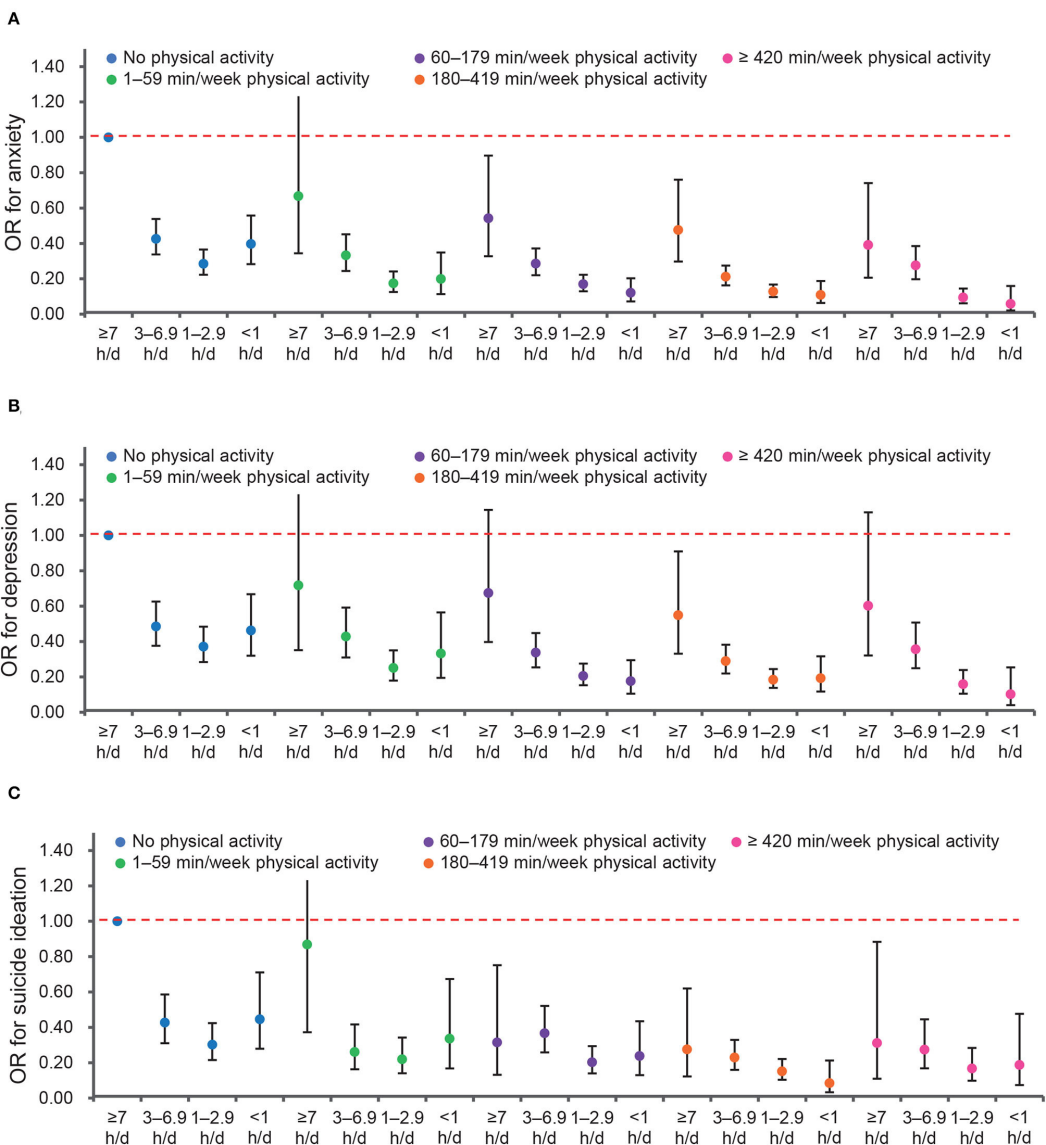
Joint associations of sedentary behavior and physical activity with emotional disorders. **(A)** Anxiety. **(B)** Depression. **(C)** Suicide ideation.

The joint analysis showed that sedentary behavior was associated with anxiety, depression, and suicide ideation in a dose-response manner (Supplementary Table 3). Even frequent physical activity (≥420 min/week) did not compensate for the risk of depression in those sitting ≥7 h/day. In contrast, physical activity was significantly associated with lower risks of anxiety and suicide ideation, even when sitting time exceeded 7 h/day (Figure 1).

The joint effect of sitting time <1 h/day and physical activity ≥ 420 min/week demonstrated the greatest effect on anxiety (AOR = 0.06, 95% CI: 0.02–0.16, *P* < 0.001), depression (AOR = 0.10, 95% CI: 0.04–0.25, *P* < 0.001), and suicide ideation (AOR = 0.19, 95% CI: 0.07–0.48, *P* < 0.001), respectively.

To examine whether the association of sedentary behavior with mental well-being was modified by physical activity, interaction terms were then introduced in the models (Supplementary Table 4). With a few exceptions, the interaction terms between sedentary behavior and physical activity were not significant, indicating that physical activity did not modify the effect. As a result, no further analysis was done by the stratum of physical activity.

### Mediation Effect

**Figure 2:**
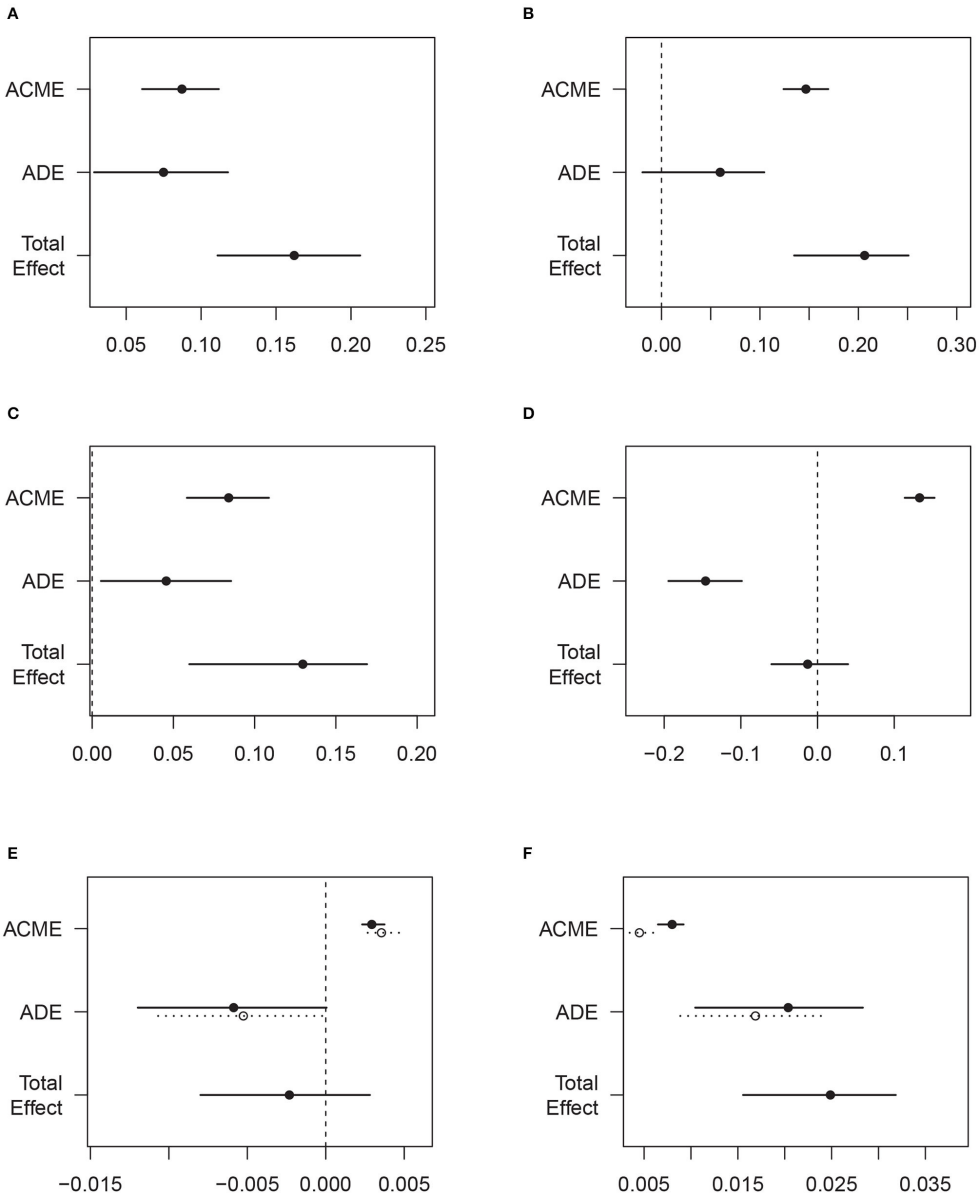
Mediation effects by sleep quality in the associations of sedentary behavior/physical activity with emotional disorders. **(A)** Sedentary behavior and anxiety. **(B)** Physical activity and anxiety. **(C)** Sedentary behavior and depression. **(D)** Physical activity and depression. **(E)** Sedentary behavior and suicide ideation. **(F)** Physical activity and suicide ideation.

To test the mediation effect of sleep quality (measured by PSQI), mediation effect analysis was conducted. According to Figure 2 and Supplementary Table 5, sleep significantly mediated 54 and 71% of the effects of sedentary behavior and physical activity on anxiety, respectively. Sleep also significantly mediated 65% of the effect of sedentary behavior on depression and 25% of the effect of physical activity on suicide ideation. Some insignificant results were also observed.

### Sensitivity Analysis

After excluding data collected in the pilot study in 2017, a total of 20,072 subjects was included in the sensitivity analysis. Supplementary Table 6 presents the multivariable-adjusted joint effect of sedentary behavior and physical activity. The effect size remained consistent in general, with some small fluctuations in AORs.

## Discussion

We investigated sedentary behavior and physical activity, and their association with mental well-being among first-year college students in China. Long sedentary time and lack of physical activity, in general, were common among college students and were even more prevalent in students with emotional distress, which can be partly explained by their impaired sleeping quality. More importantly, sedentary behavior showed a dose-response relationship with anxiety, depression, and suicide ideation, despite the level of physical activity. To our knowledge, this was among the first multi-centered study that systematically investigated the association between sedentary behavior and emotional distress in a representative sample of first-year college students in China.

Consistent with previous findings, our study found that less-sedentary behavior and more physical activity were inversely associated with the symptoms and the prevalence of anxiety, depression, and suicide ideation (22). Admittedly, there are lots of explanations for the association between physical activity with emotional distress. For example, engagement in more challenging physical activities could help to build a person's confidence and, subsequently, to decrease their depressive symptoms (23). Besides, social relationships developed from regular participation in physical activity may positively impact their mental health (24). Wagnsson et al. demonstrated that perceived sport competence plays an important mediating role in the relationship between sport participation and self-esteem (25).

When it comes to the association between sedentary behavior and mental well-being, the underlying mechanisms are still unclear. Most of the previous researchers viewed that sedentary behavior affects our mental health dependently on the lack of physical activity (15). However, our data demonstrated that physical activity did not modify the effect of sedentary behavior on mental distress, which can be backed up by Ole's findings (26). He found out that physical activity and sedentary behaviors were not associated with each other in countries with relatively low levels of physical activity. More importantly, we found that sedentary behavior was associated with anxiety, depression, and suicide ideation in a clear dose-response manner. Even frequent physical activity (≥420 min/week) did not compensate for the risk of depression in those sitting ≥7 h/day.

There are several explanations for the independent effect of sedentary behavior. First, increased sedentary time may prevent adolescents from social interactions and hence increase their risk for depression (27). This pathway can be supported by certain social/psychological theories, such as the social withdrawal hypothesis (28). Given the fact that sedentary behavior often takes place alone, it may lead to feelings of loneliness and, consequently, negatively impacts on mental health (29). Hence, higher levels of sitting time beyond standardized class hours, such as excessive screen time, could lead to social isolation and mental health problems (30). Second, a systemic inflammatory process may serve as an underlying mechanism of the association between less sedentary behavior and mental illness as well (31). A European adolescent survey demonstrated that being long-term sedentary could increase inflammatory markers, such as IL-6 among young adults (32). Last but not least, we found that sleep quality (measured by PSQI) significantly mediated 54, 65, and 25% of the effects of sedentary behavior on anxiety, depression, and suicide ideation, respectively, which was hinted by the previous findings regarding sedentary behavior and sleep problems (33).

This mediating pathway could be explained from several aspects: (1) common systemic inflammatory pathways and markers like IL-6 can be shared by anxiety and sleep problems; and (2) some sedentary behavior, such as long-standing time and excessive media exposure at night, can alter circadian rhythms and displace sleep (34, 35). Evidence also showed that being inactive may change serum melatonin levels and result in a shift in the onset of nocturnal melatonin (36).

However, even though we demonstrated that physical activity did not modify the effect of sedentary behavior, we do not encourage viewing sedentary behavior and physical inactivity separately when it comes to the explanation of impaired mental status. Our results showed that the joint effect of sitting time <1 h/day and physical activity ≥420 min/week demonstrated the greatest effect on anxiety (AOR = 0.06, *P* < 0.001), depression (AOR = 0.10, *P* < 0.001), and suicide ideation (AOR = 0.19, *P* < 0.001), respectively, revealing their synergistic effects to increase risk. The primary possibility that sedentary behavior shows a better result of the mediation effect is because sedentary behavior, including every status opposite to be sedentary, can be of more extensive meanings when compared to actual physical activity.

A primary limitation of this study is the limited generalizability among those adolescents who do not attend university. The burden of emotional distress in non-students of similar age may be different from that in students, particularly given potential socioeconomic differences. However, the gender ratio in our study participants is similar to that in the general population of similar age. According to the recent national statistics of China, the male-to-female ratio was 1:18 among people aged 15–19; and in our study, the ratio was 1:15 (15,141/13,157) (37). Second, both outcomes were in a self-reported manner, which may result in misclassification bias, especially among participants with limited literacy or misunderstanding issue. However, the reliability of the outcomes is relatively high since the studied population is all college students. The outcome indicators, PHQ-2 and GAD-2, have been proved to be widely used and reliable screening tools for depression and anxiety (38). Third, owing to the limitation of the cross-sectional study, we could not conclude on a causal relationship. Reversed causality is also possible since people with impaired mental well-being are less likely to participate in physical activities and more likely to have sedentary behaviors.

The study also has strengths. First, this was a multi-center study with a large sample size of a group of first-year college students who have just experienced similar primary and secondary education phases in China, which can offer us a relatively reliable result regarding the actual frequency and time of sedentary behavior and physical activity. Second, the measurements of anxiety and depression were performed using validated generic tools that enable comparisons across the different populations (38).

## Conclusion

In summary, the study not only provides data on sedentary behavior and physical exercise in multi-college students in China but also identified a dose-response association of sedentary behavior to emotional distress among them, suggesting this association may be independent from the effect of physical exercise and partially attributable to sleep quality. Both education practitioners and mental health providers should pay attention to the adverse psychological impacts of increased sedentary behavior, in addition to the less physical activity and impaired sleep quality.

## Data Availability Statement

The raw data supporting the conclusions of this article will be made available by the authors, without undue reservation.

## Ethics Statement

The studies involving human participants were reviewed and approved by the medical ethics committee approved this study of Xiangya Hospital, Central South University (approval number: 201709993). The patients/participants provided their written informed consent to participate in this study.

## Author Contributions

LJ drafted the manuscript. XC, MS, JH, and HL designed the study, coordinated the field survey, and critically reviewed and revised the manuscript. The other authors participated in data interpretation, review, and revision of the manuscript. All authors contributed to the article and approved the submitted version.

## Conflict of Interest

The authors declare that the research was conducted in the absence of any commercial or financial relationships that could be construed as a potential conflict of interest.
