# Supplementary material for: Association of Sedentary Behavior With Anxiety, Depression, and Suicide Ideation in College Students
Source: Front Psychiatry. 2020 Dec 11;11:566098. doi: 10.3389/fpsyt.2020.566098 (PMC7793895; doi:10.3389/fpsyt.2020.566098)
Supplement: Supplementary file 1 [file Data_Sheet_1.docx]

**Table S1. Center effect in null models**

| Outcome variable | Link function | Distribution | Level-2 variance (SE) | Level-1 variance (SE) | ICC (%) |
| --- | --- | --- | --- | --- | --- |
| Anxiety | Logit | Binomial | 0.143 (0.094) | 1.7 (0) | 7.76 |
| Depression | Logit | Binomial | 0.188 (0.125) | 1.7 (0) | 9.96 |
| Suicide ideation | Logit | Binomial | 0.221 (0.151) | 1.7 (0) | 11.50 |

GAD-2: Two-Item Generalized Anxiety Disorder Scale. PHQ-2: Two-Item Patient Health Questionnaire. SE: standard error. ICC: intra-cluster correlation coefficient.

**Table S2. Independent effect of sedentary behavior and physical activity on anxiety, depression, and suicide ideation**

| Exposure | Anxiety (GAD-2 ≥3) | | | | |  | Depression (PHQ-2 ≥3) | | | | |  | Suicide ideation | | | | |
| --- | --- | --- | --- | --- | --- | --- | --- | --- | --- | --- | --- | --- | --- | --- | --- | --- | --- |
|  | Crude estimates | |  | Adjusted estimates ^a^ | |  | Crude estimates | |  | Adjusted estimates ^a^ | |  | Crude estimates | |  | Adjusted estimates ^a^ | |
|  | OR (95% CI) | *P* |  | AOR (95% CI) | *P* |  | AOR (95% CI) | *P* |  | AOR (95% CI) | *P* |  | OR (95% CI) | *P* |  | AOR (95% CI) | *P* |
| Sedentary behavior (h/day) | | | | | | | | | | | | | | | | | |
| ≥ 7 | Ref |  |  | Ref |  |  | Ref |  |  | Ref |  |  | Ref |  |  | Ref |  |
| 3 to 6.9 | 0.42 (0.36, 0.50) | <0.001 |  | 0.47 (0.39, 0.56) | <0.001 |  | 0.47 (0.39, 0.56) | <0.001 |  | 0.52 (0.43, 0.63) | <0.001 |  | 0.47 (0.37, 0.61) | <0.001 |  | 0.54 (0.42, 0.69) | <0.001 |
| 1 to 2.9 | 0.24 (0.20, 0.29) | <0.001 |  | 0.28 (0.23, 0.34) | <0.001 |  | 0.29 (0.24, 0.35) | <0.001 |  | 0.34 (0.28, 0.42) | <0.001 |  | 0.30 (0.23, 0.39) | <0.001 |  | 0.36 (0.28, 0.47) | <0.001 |
| < 1 | 0.26 (0.20, 0.33) | <0.001 |  | 0.30 (0.23, 0.38) | <0.001 |  | 0.32 (0.25, 0.42) | <0.001 |  | 0.37 (0.29, 0.48) | <0.001 |  | 0.37 (0.27, 0.52) | <0.001 |  | 0.44 (0.31, 0.62) | <0.001 |
| Physical activity (min/week) | | | | | | | | | | | | | | | | | |
| No | Ref |  |  | Ref |  |  | Ref |  |  | Ref |  |  | Ref |  |  | Ref |  |
| 1 to 59 | 0.56 (0.48, 0.66) | <0.001 |  | 0.69 (0.58, 0.81) | <0.001 |  | 0.65 (0.55, 0.77) | <0.001 |  | 0.78 (0.66, 0.93) | 0.006 |  | 0.57 (0.45, 0.74) | <0.001 |  | 0.69 (0.54, 0.89) | 0.004 |
| 60 to 179 | 0.53 (0.47, 0.60) | <0.001 |  | 0.61 (0.53, 0.69) | <0.001 |  | 0.54 (0.47, 0.61) | <0.001 |  | 0.62 (0.54, 0.71) | <0.001 |  | 0.62 (0.52, 0.74) | <0.001 |  | 0.71 (0.59, 0.86) | <0.001 |
| 180 to 419 | 0.42 (0.37, 0.47) | <0.001 |  | 0.46 (0.40, 0.53) | <0.001 |  | 0.49 (0.43, 0.55) | <0.001 |  | 0.55 (0.47, 0.63) | <0.001 |  | 0.41 (0.34, 0.50) | <0.001 |  | 0.47 (0.39, 0.58) | <0.001 |
| ≥ 420 | 0.43 (0.35, 0.53) | <0.001 |  | 0.45 (0.37, 0.56) | <0.001 |  | 0.51 (0.41, 0.62) | <0.001 |  | 0.55 (0.45, 0.69) | <0.001 |  | 0.49 (0.37, 0.66) | <0.001 |  | 0.55 (0.41, 0.74) | <0.001 |

GAD-2: Two-Item Generalized Anxiety Disorder Scale. PHQ-2: Two-Item Patient Health Questionnaire. OR: odds ratio. AOR: adjusted odds ratio. CI: confidence interval. Ref: reference group.

^a^ Adjusted for level-1 confounders (region, age, body mass index, gender, ethnicity, annual family income, physical activities, sedentary activities, alcohol drinking) and level-2 confounder (year of enrolment) with random intercepts (university). Sedentary behavior and physical activity were simultaneously included in adjusted model.

**Table S3. Joint effect of sedentary behavior and physical activity on anxiety, depression, and suicide ideation**

| Sedentary  behavior  (h/day) | Physical  activity  (min/week) | Anxiety (GAD-2 ≥3) | | | | |  | Depression (PHQ-2 ≥3) | | | | |  | Suicide ideation | | | | |
| --- | --- | --- | --- | --- | --- | --- | --- | --- | --- | --- | --- | --- | --- | --- | --- | --- | --- | --- |
|  |  | Crude estimates | |  | Adjusted estimates ^a^ | |  | Crude estimates | |  | Adjusted estimates ^a^ | |  | Crude estimates | |  | Adjusted estimates ^a^ | |
|  |  | OR (95% CI) | *P* |  | AOR (95% CI) | *P* |  | OR (95% CI) | *P* |  | AOR (95% CI) | *P* |  | OR (95% CI) | *P* |  | AOR (95% CI) | *P* |
| ≥ 7 | No | Ref |  |  | Ref |  |  | Ref |  |  | Ref |  |  | Ref |  |  | Ref |  |
| 3 to 6.9 | No | 0.42 (0.33, 0.53) | <0.001 |  | 0.43 (0.34, 0.54) | <0.001 |  | 0.47 (0.37, 0.61) | <0.001 |  | 0.49 (0.38, 0.63) | <0.001 |  | 0.41 (0.30, 0.57) | <0.001 |  | 0.43 (0.31, 0.59) | <0.001 |
| 1 to 2.9 | No | 0.28 (0.22, 0.36) | <0.001 |  | 0.29 (0.22, 0.37) | <0.001 |  | 0.36 (0.28, 0.47) | <0.001 |  | 0.37 (0.28, 0.48) | <0.001 |  | 0.30 (0.21, 0.42) | <0.001 |  | 0.30 (0.22, 0.42) | <0.001 |
| < 1 | No | 0.41 (0.29, 0.57) | <0.001 |  | 0.40 (0.28, 0.56) | <0.001 |  | 0.47 (0.33, 0.68) | <0.001 |  | 0.46 (0.32, 0.67) | <0.001 |  | 0.45 (0.28, 0.72) | <0.001 |  | 0.45 (0.28, 0.71) | <0.001 |
| ≥ 7 | 1 to 59 | 0.63 (0.34, 1.18) | 0.153 |  | 0.67 (0.34, 1.29) | 0.231 |  | 0.69 (0.34, 1.39) | 0.296 |  | 0.72 (0.35, 1.47) | 0.362 |  | 0.85 (0.37, 1.94) | 0.698 |  | 0.87 (0.37, 2.02) | 0.744 |
| 3 to 6.9 | 1 to 59 | 0.30 (0.22, 0.40) | <0.001 |  | 0.33 (0.24, 0.45) | <0.001 |  | 0.38 (0.27, 0.52) | <0.001 |  | 0.43 (0.31, 0.59) | <0.001 |  | 0.23 (0.14, 0.36) | <0.001 |  | 0.26 (0.16, 0.42) | <0.001 |
| 1 to 2.9 | 1 to 59 | 0.16 (0.12, 0.21) | <0.001 |  | 0.17 (0.13, 0.24) | <0.001 |  | 0.22 (0.16, 0.31) | <0.001 |  | 0.25 (0.18, 0.35) | <0.001 |  | 0.19 (0.12, 0.30) | <0.001 |  | 0.22 (0.14, 0.34) | <0.001 |
| < 1 | 1 to 59 | 0.18 (0.11, 0.31) | <0.001 |  | 0.20 (0.11, 0.35) | <0.001 |  | 0.30 (0.17, 0.50) | <0.001 |  | 0.33 (0.19, 0.56) | <0.001 |  | 0.29 (0.15, 0.59) | <0.001 |  | 0.34 (0.17, 0.67) | 0.002 |
| ≥ 7 | 60 to 179 | 0.55 (0.34, 0.90) | 0.017 |  | 0.54 (0.33, 0.90) | 0.017 |  | 0.67 (0.39, 1.13) | 0.131 |  | 0.67 (0.40, 1.14) | 0.144 |  | 0.32 (0.13, 0.76) | 0.001 |  | 0.31 (0.13, 0.75) | 0.009 |
| 3 to 6.9 | 60 to 179 | 0.27 (0.21, 0.35) | <0.001 |  | 0.29 (0.22, 0.37) | <0.001 |  | 0.31 (0.24, 0.41) | <0.001 |  | 0.34 (0.25, 0.45) | <0.001 |  | 0.33 (0.24, 0.47) | <0.001 |  | 0.37 (0.26, 0.52) | <0.001 |
| 1 to 2.9 | 60 to 179 | 0.16 (0.12, 0.21) | <0.001 |  | 0.17 (0.13, 0.22) | <0.001 |  | 0.19 (0.14, 0.25) | <0.001 |  | 0.21 (0.15, 0.28) | <0.001 |  | 0.18 (0.13, 0.27) | <0.001 |  | 0.20 (0.14, 0.29) | <0.001 |
| < 1 | 60 to 179 | 0.12 (0.07, 0.19) | <0.001 |  | 0.12 (0.07, 0.20) | <0.001 |  | 0.17 (0.10, 0.28) | <0.001 |  | 0.18 (0.11, 0.29) | <0.001 |  | 0.22 (0.12, 0.41) | <0.001 |  | 0.24 (0.13, 0.43) | <0.001 |
| ≥ 7 | 180 to 419 | 0.49 (0.31, 0.78) | 0.002 |  | 0.48 (0.30, 0.76) | 0.002 |  | 0.54 (0.33, 0.89) | 0.016 |  | 0.55 (0.33, 0.91) | 0.020 |  | 0.28 (0.12, 0.62) | 0.002 |  | 0.28 (0.12, 0.62) | 0.002 |
| 3 to 6.9 | 180 to 419 | 0.21 (0.16, 0.27) | <0.001 |  | 0.21 (0.16, 0.27) | <0.001 |  | 0.27 (0.21, 0.36) | <0.001 |  | 0.29 (0.22, 0.38) | <0.001 |  | 0.21 (0.15, 0.30) | <0.001 |  | 0.23 (0.16, 0.33) | <0.001 |
| 1 to 2.9 | 180 to 419 | 0.12 (0.09, 0.16) | <0.001 |  | 0.13 (0.10, 0.17) | <0.001 |  | 0.17 (0.13, 0.23) | <0.001 |  | 0.18 (0.14, 0.24) | <0.001 |  | 0.14 (0.09, 0.20) | <0.001 |  | 0.15 (0.10, 0.22) | <0.001 |
| < 1 | 180 to 419 | 0.10 (0.06, 0.18) | <0.001 |  | 0.11 (0.06, 0.19) | <0.001 |  | 0.18 (0.11, 0.29) | <0.001 |  | 0.19 (0.12, 0.32) | <0.001 |  | 0.07 (0.03, 0.19) | <0.001 |  | 0.08 (0.03, 0.21) | <0.001 |
| ≥ 7 | ≥ 420 | 0.39 (0.21, 0.72) | 0.002 |  | 0.39 (0.21, 0.74) | 0.004 |  | 0.58 (0.31, 1.09) | 0.089 |  | 0.60 (0.32, 1.13) | 0.114 |  | 0.30 (0.10, 0.83) | 0.021 |  | 0.31 (0.11, 0.88) | 0.028 |
| 3 to 6.9 | ≥ 420 | 0.28 (0.20, 0.39) | <0.001 |  | 0.28 (0.20, 0.39) | <0.001 |  | 0.34 (0.24, 0.49) | <0.001 |  | 0.36 (0.25, 0.51) | <0.001 |  | 0.26 (0.16, 0.42) | <0.001 |  | 0.27 (0.17, 0.45) | <0.001 |
| 1 to 2.9 | ≥ 420 | 0.09 (0.06, 0.14) | <0.001 |  | 0.09 (0.06, 0.14) | <0.001 |  | 0.15 (0.10, 0.23) | <0.001 |  | 0.16 (0.10, 0.24) | <0.001 |  | 0.15 (0.09, 0.26) | <0.001 |  | 0.17 (0.10, 0.28) | <0.001 |
| < 1 | ≥ 420 | 0.06 (0.02, 0.15) | <0.001 |  | 0.06 (0.02, 0.16) | <0.001 |  | 0.10 (0.04, 0.24) | <0.001 |  | 0.10 (0.04, 0.25) | <0.001 |  | 0.17 (0.07, 0.44) | <0.001 |  | 0.19 (0.07, 0.48) | <0.001 |

GAD-2: Two-Item Generalized Anxiety Disorder Scale. PHQ-2: Two-Item Patient Health Questionnaire. OR: odds ratio. AOR: adjusted odds ratio. CI: confidence interval. Ref: reference group.

^a^ Adjusted for level-1 confounders (region, age, body mass index, gender, ethnicity, annual family income, physical activities, sedentary activities, alcohol drinking) and level-2 confounder (year of enrolment) with random intercepts (university).

**Table S4. Modification effect of physical activity and sedentary behavior on anxiety, depression, and suicide ideation**

| Exposure | Anxiety (GAD-2 ≥3) | | | | |  | Depression (PHQ-2 ≥3) | | | | |  | Suicide ideation | | | | |
| --- | --- | --- | --- | --- | --- | --- | --- | --- | --- | --- | --- | --- | --- | --- | --- | --- | --- |
|  | Crude estimates | |  | Adjusted estimates ^a^ | |  | Crude estimates | |  | Adjusted estimates ^a^ | |  | Crude estimates | |  | Adjusted estimates ^a^ | |
|  | *β* (SE) | *P* |  | *β* (SE) | *P* |  | *β* (SE) | *P* |  | *β* (SE) | *P* |  | *β* (SE) | *P* |  | *β* (SE) | *P* |
| Sedentary behavior (h/day) | | | | | | | | | | | | | | | | | |
| [a1] ≥ 7 | Ref |  |  | Ref |  |  | Ref |  |  | Ref |  |  | Ref |  |  | Ref |  |
| [a1] 3 to 6.9 | –0.88 (0.12) | <0.001 |  | -0.85 (0.12) | <0.001 |  | –0.75 (0.13) | <0.001 |  | –0.72 (0.13) | <0.001 |  | –0.89 (0.16) | <0.001 |  | -0.85 (2.16) | <0.001 |
| [a1] 1 to 2.9 | –1.28 (0.13) | <0.001 |  | -1.25 (0.13) | <0.001 |  | –1.01 (0.14) | <0.001 |  | –0.99 (0.14) | <0.001 |  | –1.22 (0.17) | <0.001 |  | -1.20 (1.17) | <0.001 |
| [a1] < 1 | –0.91 (0.17) | <0.001 |  | -0.92 (0.17) | <0.001 |  | –0.76 (0.19) | <0.001 |  | –0.77 (0.19) | <0.001 |  | –0.79 (0.24) | <0.001 |  | -0.81 (0.24) | <0.001 |
| Physical activity (min/week) | | | | | | | | | | | | | | | | | |
| [b1] No | Ref |  |  | Ref |  |  | Ref |  |  | Ref |  |  | Ref |  |  | Ref |  |
| [b2] 1 to 59 | –0.46 (0.33) | 0.165 |  | -0.40 (0.34) | 0.231 |  | –0.38 (0.36) | 0.291 |  | –0.33 (0.36) | 0.362 |  | –0.16 (0.42) | 0.702 |  | -0.14 (.43) | 0.744 |
| [b3] 60 to 179 | –0.61 (0.25) | 0.017 |  | -0.61 (0.26) | 0.017 |  | –0.41 (0.27) | 0.127 |  | –0.39 (0.27) | 0.144 |  | –1.15 (0.44) | 0.009 |  | -1.16 (.44) | 0.009 |
| [b4] 180 to 419 | –0.73 (0.24) | 0.002 |  | -0.74 (0.24) | 0.002 |  | –0.62 (0.26) | 0.016 |  | –0.60 (0.26) | 0.02 |  | –1.29 (0.41) | 0.002 |  | -1.29 (.41) | 0.002 |
| [b5] ≥ 420 | –0.95 (0.32) | 0.003 |  | -0.94 (0.33) | 0.004 |  | –0.54 (0.32) | 0.089 |  | –0.51 (0.32) | 0.114 |  | –1.22 (0.53) | 0.021 |  | -1.17 (.53) | 0.028 |
| Interaction term |  |  |  |  |  |  |  |  |  |  |  |  |  |  |  |  |  |
| [a1] or [b1] | Ref |  |  | Ref |  |  | Ref |  |  | Ref |  |  | Ref |  |  | Ref |  |
| [a2] and [b2] | 0.11 (0.35) | 0.758 |  | 0.16 (0.36) | 0.666 |  | 0.15 (0.38) | 0.693 |  | 0.21 (0.39) | 0.591 |  | –0.44 (0.47) | 0.349 |  | -0.35 (2.48) | 0.460 |
| [a2] and [b3] | 0.17 (0.27) | 0.537 |  | 0.21 (0.27) | 0.435 |  | –0.01 (0.29) | 0.983 |  | 0.03 (0.29) | 0.917 |  | 0.93 (0.46) | 0.043 |  | 1.01 (2.46) | 0.030 |
| [a2] and [b4] | 0.02 (0.25) | 0.953 |  | 0.04 (0.26) | 0.868 |  | 0.07 (0.27) | 0.806 |  | 0.08 (0.27) | 0.761 |  | 0.62 (0.43) | 0.152 |  | 0.67 (2.44) | 0.124 |
| [a2] and [b5] | 0.54 (0.35) | 0.124 |  | 0.50 (0.36) | 0.156 |  | 0.22 (0.35) | 0.529 |  | 0.20 (0.35) | 0.576 |  | 0.76 (0.57) | 0.186 |  | 0.72 (2.57) | 0.207 |
| [a3] and [b2] | –0.13 (0.36) | 0.718 |  | -0.09 (0.37) | 0.805 |  | –0.11 (0.39) | 0.778 |  | –0.06 (0.39) | 0.877 |  | –0.27 (0.47) | 0.559 |  | -0.18 (1.47) | 0.705 |
| [a3] and [b3] | 0.04 (0.28) | 0.890 |  | 0.09 (0.28) | 0.738 |  | –0.24 (0.29) | 0.401 |  | –0.20 (0.29) | 0.502 |  | 0.67 (0.47) | 0.153 |  | 0.76 (1.47) | 0.108 |
| [a3] and [b4] | –0.11 (0.26) | 0.684 |  | -0.07 (0.26) | 0.804 |  | –0.13 (0.28) | 0.645 |  | –0.10 (0.28) | 0.711 |  | 0.51 (0.44) | 0.244 |  | 0.60 (1.44) | 0.178 |
| [a3] and [b5] | –0.16 (0.38) | 0.676 |  | -0.17 (0.38) | 0.660 |  | –0.35 (0.37) | 0.346 |  | –0.34 (0.37) | 0.354 |  | 0.55 (0.58) | 0.343 |  | 0.58 (1.59) | 0.325 |
| [a4] and [b2] | –0.36 (0.45) | 0.420 |  | -0.29 (0.45) | 0.521 |  | –0.08 (0.46) | 0.867 |  | 0.00 (0.46) | 0.999 |  | –0.27 (0.56) | 0.630 |  | -0.14 (0.57) | 0.805 |
| [a4] and [b3] | –0.63 (0.38) | 0.091 |  | -0.58 (0.38) | 0.126 |  | –0.63 (0.38) | 0.103 |  | –0.57 (0.39) | 0.138 |  | 0.44 (0.55) | 0.431 |  | 0.53 (0.55) | 0.340 |
| [a4] and [b4] | –0.65 (0.37) | 0.081 |  | -0.55 (0.37) | 0.141 |  | –0.35 (0.37) | 0.340 |  | –0.27 (0.37) | 0.459 |  | –0.51 (0.64) | 0.421 |  | -0.38 (0.64) | 0.557 |
| [a4] and [b5] | –0.99 (0.61) | 0.104 |  | -0.99 (0.62) | 0.109 |  | –1.05 (0.57) | 0.067 |  | –1.01 (0.57) | 0.079 |  | 0.25 (0.72) | 0.724 |  | 0.30 (0.73) | 0.679 |

GAD-2: Two-Item Generalized Anxiety Disorder Scale. PHQ-2: Two-Item Patient Health Questionnaire. *β*: regression coefficient. SE: standard error. Ref: reference group.

^a^ Adjusted for level-1 confounders (region, age, body mass index, gender, ethnicity, annual family income, physical activities, sedentary activities, alcohol drinking) and level-2 confounder (year of enrolment) with random intercepts (university).

**Table S5. Mediation effect of sleep quality**

|  | Anxiety | |  | Depression | |  | Suicide ideation | |
| --- | --- | --- | --- | --- | --- | --- | --- | --- |
| Exposure | Proportion mediated (95% CI) | *P* |  | Proportion mediated by sleep | *P* |  | Proportion mediated (95% CI) | *P* |
| Sedentary behavior | 0.54 (0.38, 0.74) | <0.001 |  | 0.65 (0.43, 0.93) | <0.001 |  | –0.61 (–5.64, 4.67) | 0.56 |
| Physical activity | 0.71 (0.55, 1.17) | <0.001 |  | –2.24 (–29.3, 32.0) | 0.84 |  | 0.25 (0.18, 0.41) | <0.001 |

**Table S6. Sensitivity analysis by excluding 2017 data**

| Sedentary behavior  (h/day) | Physical activity  (min/week) | Anxiety (GAD-2 ≥3) | |  | Depression (PHQ-2 ≥3) | |  | Suicide ideation | |
| --- | --- | --- | --- | --- | --- | --- | --- | --- | --- |
|  |  | AOR (95% CI) | *P* |  | AOR (95% CI) | *P* |  | AOR (95% CI) | *P* |
| ≥ 7 | No | Ref |  |  | Ref |  |  | Ref |  |
| 3 to 6.9 | No | 0.41 (0.32, 0.52) | <0.001 |  | 0.48 (0.36, 0.63) | <0.001 |  | 0.43 (0.31, 0.59) | <0.001 |
| 1 to 2.9 | No | 0.26 (0.20, 0.34) | <0.001 |  | 0.36 (0.27, 0.48) | <0.001 |  | 0.28 (0.20, 0.40) | <0.001 |
| < 1 | No | 0.39 (0.27, 0.55) | <0.001 |  | 0.47 (0.32, 0.69) | 0.0001 |  | 0.44 (0.27, 0.71) | 0.0008 |
| ≥ 7 | 1 to 59 | 0.70 (0.32, 1.51) | 0.360 |  | 0.79 (0.33, 1.86) | 0.590 |  | 0.85 (0.31, 2.31) | 0.752 |
| 3 to 6.9 | 1 to 59 | 0.34 (0.24, 0.48) | <0.001 |  | 0.42 (0.29, 0.62) | <0.001 |  | 0.26 (0.15, 0.45) | <0.001 |
| 1 to 2.9 | 1 to 59 | 0.16 (0.11, 0.23) | <0.001 |  | 0.27 (0.19, 0.41) | <0.001 |  | 0.23 (0.14, 0.38) | <0.001 |
| < 1 | 1 to 59 | 0.18 (0.09, 0.35) | <0.001 |  | 0.35 (0.18, 0.66) | 0.0011 |  | 0.38 (0.17, 0.82) | 0.014 |
| ≥ 7 | 60 to 179 | 0.52 (0.30, 0.89) | 0.017 |  | 0.65 (0.36, 1.16) | 0.147 |  | 0.22 (0.08, 0.64) | 0.005 |
| 3 to 6.9 | 60 to 179 | 0.28 (0.21, 0.37) | <0.001 |  | 0.36 (0.26, 0.48) | <0.001 |  | 0.33 (0.22, 0.48) | <0.001 |
| 1 to 2.9 | 60 to 179 | 0.16 (0.12, 0.21) | <0.001 |  | 0.24 (0.18, 0.33) | <0.001 |  | 0.21 (0.14, 0.31) | <0.001 |
| < 1 | 60 to 179 | 0.10 (0.05, 0.18) | <0.001 |  | 0.19 (0.11, 0.34) | <0.001 |  | 0.29 (0.16, 0.54) | <0.001 |
| ≥ 7 | 180 to 419 | 0.44 (0.26, 0.72) | 0.001 |  | 0.57 (0.33, 0.98) | 0.044 |  | 0.30 (0.13, 0.68) | 0.004 |
| 3 to 6.9 | 180 to 419 | 0.21 (0.16, 0.27) | <0.001 |  | 0.32 (0.23, 0.42) | <0.001 |  | 0.21 (0.15, 0.31) | <0.001 |
| 1 to 2.9 | 180 to 419 | 0.12 (0.09, 0.17) | <0.001 |  | 0.20 (0.15, 0.28) | <0.001 |  | 0.14 (0.09, 0.21) | <0.001 |
| < 1 | 180 to 419 | 0.12 (0.07, 0.21) | <0.001 |  | 0.24 (0.15, 0.41) | <0.001 |  | 0.10 (0.04, 0.25) | <0.001 |
| ≥ 7 | ≥ 420 | 0.46 (0.24, 0.89) | 0.0209 |  | 0.81 (0.42, 1.55) | 0.525 |  | 0.36 (0.13, 1.04) | 0.058 |
| 3 to 6.9 | ≥ 420 | 0.28 (0.20, 0.40) | <0.001 |  | 0.39 (0.27, 0.58) | <0.001 |  | 0.25 (0.15, 0.43) | <0.001 |
| 1 to 2.9 | ≥ 420 | 0.10 (0.06, 0.15) | <0.001 |  | 0.18 (0.12, 0.28) | <0.001 |  | 0.16 (0.09, 0.29) | <0.001 |
| < 1 | ≥ 420 | 0.07 (0.02, 0.19) | <0.001 |  | 0.13 (0.05, 0.34) | <0.001 |  | 0.17 (0.06, 0.49) | <0.001 |

GAD-2: Two-Item Generalized Anxiety Disorder Scale. PHQ-2: Two-Item Patient Health Questionnaire. AOR: adjusted odds ratio. CI: confidence interval. Ref: reference group.

^a^ Adjusted for level-1 confounders (region, age, body mass index, gender, ethnicity, annual family income, physical activities, sedentary activities, alcohol drinking) and level-2 confounder (year of enrolment) with random intercepts (university).
